# The Role of Gut Microbiota-Bile Acids Axis in the Progression of Non-alcoholic Fatty Liver Disease

**DOI:** 10.3389/fmicb.2022.908011

**Published:** 2022-06-27

**Authors:** Yiming Ni, Mengna Lu, Yuan Xu, Qixue Wang, Xinyi Gu, Ying Li, Tongxi Zhuang, Chenyi Xia, Ting Zhang, Xiao-jun Gou, Mingmei Zhou

**Affiliations:** ^1^Institute for Interdisciplinary Medicine Sciences, Shanghai University of Traditional Chinese Medicine, Shanghai, China; ^2^Central Laboratory, Baoshan District Hospital of Integrated Traditional Chinese and Western Medicine of Shanghai, Shanghai University of Traditional Chinese Medicine, Shanghai, China; ^3^School of Pharmacy, Shanghai University of Traditional Chinese Medicine, Shanghai, China; ^4^School of Pharmacy, Shaanxi University of Traditional Chinese Medicine, Xianyang, China; ^5^Shanghai Frontiers Science Center of Traditional Chinese Medicine Chemical Biology, Institute of Interdisciplinary Integrative Medicine Research, Shanghai University of Traditional Chinese Medicine, Shanghai, China; ^6^Department of Physiology, School of Basic Medical Sciences, Shanghai University of Traditional Chinese Medicine, Shanghai, China

**Keywords:** non-alcoholic fatty liver disease (NAFLD), bile acids, gut microbiota, gut microbiota-bile acids axis, disease progression

## Abstract

Non-alcoholic fatty liver disease (NAFLD), an emerging global health problem affecting 25–30% of the total population, refers to excessive lipid accumulation in the liver accompanied by insulin resistance (IR) without significant alcohol intake. The increasing prevalence of NAFLD will lead to an increasing number of cirrhosis patients, as well as hepatocellular carcinoma (HCC) requiring liver transplantation, while the current treatments for NAFLD and its advanced diseases are suboptimal. Accordingly, it is necessary to find signaling pathways and targets related to the pathogenesis of NAFLD for the development of novel drugs. A large number of studies and reviews have described the critical roles of bile acids (BAs) and their receptors in the pathogenesis of NAFLD. The gut microbiota (GM), whose composition varies between healthy and NAFLD patients, promotes the transformation of more than 50 secondary bile acids and is involved in the pathophysiology of NAFLD through the GM-BAs axis. Correspondingly, BAs inhibit the overgrowth of GM and maintain a healthy gut through their antibacterial effects. Here we review the biosynthesis, enterohepatic circulation, and major receptors of BAs, as well as the relationship of GM, BAs, and the pathogenesis of NAFLD in different disease progression. This article also reviews several therapeutic approaches for the management and prevention of NAFLD targeting the GM-BAs axis.

## Introduction

Non-alcoholic fatty liver disease (NAFLD), an emerging global health problem affecting 25–30% of the total population, refers to excessive lipid accumulation in the liver accompanied by insulin resistance (IR) without significant alcohol intake (Caussy et al., 2019; Hrncir et al., 2021). NAFLD, characterized by steatosis, necroinflammatory changes, and varying degrees of liver fibrosis, is the most common cause of chronic liver disease in developed countries (Gottlieb and Canbay, 2019; Ji et al., 2020). Closely related to atherosclerosis, metabolic syndrome, obesity, diabetes, coronary heart disease, and other metabolic diseases, NAFLD’s disease spectrum includes simple steatosis, namely non-alcoholic fatty liver, irreversible non-alcoholic steatohepatitis (NASH), more serious morbid state, like liver fibrosis and cirrhosis, and hepatocellular carcinoma (HCC) (Bhatia et al., 2012; Koliaki et al., 2015; Younossi et al., 2016a; Hrncir et al., 2021). It is estimated that more than 60% of NAFLD patients undergoing liver biopsy have NASH, and about 40% of NASH patients show noticeable symptoms of fibrosis (Younossi et al., 2016b). Approximately 22% of patients with bridging fibrosis (F3) go on to develop cirrhosis, and those with advanced fibrosis are more likely to develop HCC (Sanyal et al., 2019). The increasing prevalence of the disease will be accompanied by an increasing number of patients with cirrhosis and HCC requiring liver transplantation (Friedman et al., 2018). Therefore, discovering an effective NAFLD pharmacotherapy will be a challenge in the field of hepatology.

Gut microbiota (GM), considered as a special organ involved in the pathophysiology of NAFLD, is being intensively studied using various high-throughput techniques (Rinella, 2015). GM in NAFLD patients is dominated by *Firmicutes, Bacteroidetes, Proteobacteria*, and *Actinobacteria* at the phylum level (Loomba et al., 2017). Furthermore, *Eubacterium rectale, Bacteroides vulgatus*, and *Escherichia coli* were the most abundant at the species level (Loomba et al., 2017). GM can be regarded as an “endocrine organ” that regulates host physiological functions (Ridlon and Bajaj, 2015). The role of GM in the progression of NAFLD is critical for exploring its pathophysiology, identifying therapeutic targets and pathways, and subsequent appropriate therapy (Aron-Wisnewsky et al., 2020b).

Primary bile acids (BAs) are synthesized from cholesterol firstly in the liver and account for about half of the organic constituent of bile. Once primary BAs enter the gastrointestinal tract, over 50 secondary BAs are formed (Gottlieb and Canbay, 2019; Winston and Theriot, 2020). In other words, the chemical diversity of the BA pool relies on the mutual efforts of the primary BAs-producing host and the secondary BAs-producing GM. BAs in turn have antibacterial effects against certain bacteria (Winston and Theriot, 2020). Numerous studies have shown that BAs act as signaling molecules to regulate glucose, lipid and energy metabolism and the inflammatory response by mediating the intensive connection between the liver and the gut (Mouzaki et al., 2016; Jia et al., 2018; Yamada et al., 2018; Jia and Jeon, 2019; Sun et al., 2019; Sydor et al., 2020; Shao et al., 2021; Simbrunner et al., 2021; Wu et al., 2021). The composition of BAs in NAFLD patients differs from that of healthy controls, and BAs can prevent NAFLD progression by activating BAs receptors (Gottlieb and Canbay, 2019). By regulating or detecting of biomarkers on the “GM-BAs” axis, it can be helpful for the prevention, treatment and diagnosis of some diseases, including NAFLD, diarrhea-irritable bowel syndrome, polycystic ovary, etc.

In this review, we review the bio-synthesis, transport, and major receptors of BAs, the interactions between BAs and the GM, and elucidate the function of BAs as signaling molecules in the gut-liver axis, focusing on their roles in the pathogenesis of each stage of the NAFLD progression. Several management and preventive approaches for NAFLD targeting the GM-BAs axis have been proposed (Jia et al., 2018).

## Bile Acids Act as Signal Molecules Between the Gut and Liver

The enterohepatic circulation of BAs affects diverse metabolic and immune functions (Willis et al., 2020). BAs are synthesized in the liver, while GM is responsible for the formation of different BAs biomolecular structures *via* bacterial biotransformations, including deconjugation, dehydroxylation, oxidation, desulfation, dehydrogenation, and epimerization (Kang et al., 2019; Poland and Flynn, 2021; Wu et al., 2021).

### Fates and Functions of Bile Acids

#### The Enterohepatic Circulation of Bile Acids

**FIGURE 1:**
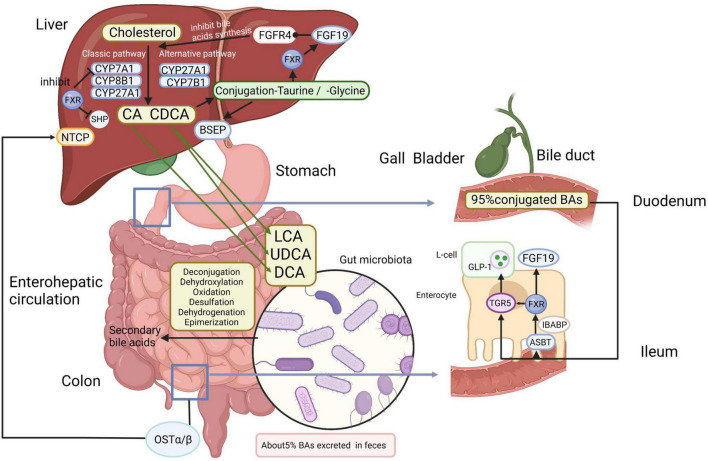
Bile acids (Bas) metabolism and enterohepatic circulation. In the liver, primary BAs (CA and CDCA) are synthesized by enzymatic catalysis from cholesterol. CYP7A1 initiates the classical synthesis pathway and the ratio of CA/CDCA is regulated by CYP8B1. CYP27A1 and CYP7B1 are mainly responsible for the alternative synthesis pathway. Then conjugated CA/CDCA with taurine or glycine are excreted across the canalicular (apical) membrane into the bile by the BSEP and stored in the gallbladder before being released to the duodenum, where primary BAs are transformed into secondary BAs by gut microbiota *via* deconjugation, dehydroxylation, oxidation, desulfation, dehydrogenation, and epimerization. Most BAs are reabsorbed in the ileum by the ASBT and then connected to the IBABP. After that, BAs are transported to the portal vein *via* OSTα/β and are taken up mainly by NTCP. In enteroendocrine cells, FXR enables TGR5 to induce the secretion of GLP-1 in intestinal L-cells. The combination of BAs to FXR not only inhibits the FXR-SHP pathway, accelerating lipid synthesis, but also induces FGF19 in the liver and ilea, and FGF19 binds to the FGFR4 on the hepatocytes, inhibiting BA synthesis. Approximately 5% of BAs are lost in fecal excretion. BAs, bile acids; CA, cholic acid; CDCA, chenodeoxycholic acid; CYP7A1, cholesterol 7α-hydroxylase; CYP8B1, sterol 12α-hydroxylase; CYP27A1, sterol 27-hydroxylase; CYP7B1, oxysterol 7α-hydroxylase; BSEP, bile salt export pump; ASBT, apical sodium-dependent bile acid transporter; IBABP, ileal bile acid-binding protein; OST, organic solute transporter; NTCP, sodium taurocholate co-transporting polypeptide; FXR, farnesoid X receptor; TGR5, Takeda G protein-coupled receptor 5; GLP-1, glucagon-like peptide-1; SHP, small heterodimer partner; FGF19, fibroblast growth factor 15/19; FGFR4, fibroblast growth factor receptor 4. Created with BioRender.com.

The liver is located at the intersection point of the portal blood flow from the intestinal circulation to peripheral organs (Tilg et al., 2016). The rate-limiting enzyme cholesterol 7α-hydroxylase (CYP7A1) in the liver initiates the classical synthesis pathway that converts cholesterol to 7α-hydroxycholesterol. 7α-hydroxycholesterol is then transformed to 7α-hydroxy-4-cholesten-3-one (C4), an intermediate for the *de novo* synthesis of primary BAs, cholic acid (CA) and chenodeoxycholic acid (CDCA) (Chiang and Ferrell, 2018, 2020). Sterol 12α-hydroxylase (CYP8B1) hydroxylates C4 and produces CA to regulate the CA/CDCA ratio, as loss of CYP8B1 results in CDCA production (Chiang and Ferrell, 2020). The alternative synthesis pathway is initiated in the mitochondrial membrane by sterol 27-hydroxylase (CYP27A1) and oxysterol 7α-hydroxylase (CYP7B1). CYP27A1, expressed extra-hepatically in a variety of tissues, catalyzes the production of CDCA or muricholic acids (in mice only), while CYP7B1 is responsible for hydroxylation of 27-hydroxycholesterol and 25-hydroxycholesterol (Ridlon and Bajaj, 2015; Chiang and Ferrell, 2018; Ji et al., 2020). Feedback inhibition of the classic bile acid synthetic enzymes CYP7A1, CYP8B1, and CYP27A1 by BAs is a well-established mechanism that reduces primary BA synthesis in response to an expanded bile acid pool or cholestasis with obviously elevated bile acid concentrations (Copple and Li, 2016). Most primary BAs are conjugated to taurine or glycine so as to enhance solubility and reduce toxicity (Chiang and Ferrell, 2019). Then conjugated CA/CDCA are excreted into the bile *via* the bile salt export pump (BSEP) across the canalicular (apical) membrane and stored in the cholecyst (Wahlström et al., 2016). Cholecystokinin secreted by the duodenum after meals, prompts gallbladder contraction, and releases BAs into the gut (Li and Chiang, 2014). After reaching the terminal ileum, approximately 95% of BAs are assimilated from the intestine by the apical sodium-dependent bile acid transporter (ASBT), a protein located in the brush border of the enterocyte (Wahlström et al., 2016; Jia et al., 2018). These BAs are associated to the ileal bile acid-binding protein (IBABP), which regulates the transportation of BAs from the intestinal epithelium to the basolateral membrane, where the heteromeric organic solute transporter (OST) promotes the transport of BAs to the portal vein (Jia et al., 2018). In other words, BAs are recirculated to the liver through the portal vein and are mainly taken up by the sodium taurocholate co-transporting polypeptide (NTCP), which is responsible for the absorption of most of the conjugated BAs (Jia et al., 2018). The mechanism through which BAs can return to the liver again through the portal vein is called “enterohepatic circulation” (Figure 1), and exerts noteworthy physiological functions in feedback inhibition of BA synthesis and regulation of systemic lipid homeostasis (Chiang, 2009; Marra and Svegliati-Baroni, 2018). Small amounts of BAs (<5%) escape the uptake mediated by protein transporters in the ileum before entering the colon (Poland and Flynn, 2021). A fraction of primary BAs is deconjugated and dehydroxylated by anaerobic GM in the distal small intestine and colon, resulting in the production of secondary BAs, lithocholic acid (LCA) and deoxycholic acid (DCA), which can be passively uptaken from the large intestine or excreted in the feces (Kwong et al., 2015; Arab et al., 2017).

#### Signaling Receptors of Bile Acids

Bile acids were under study over the past several decades and were well-recognized to function as endogenous signaling molecules to regulate metabolic processes, such as liver function and cholesterol metabolism by diverse receptors (de Aguiar Vallim et al., 2013; Arab et al., 2017). Specific receptors mediate the regulation of BAs, including the members of the nuclear receptor superfamily and G protein-coupled receptor superfamily, such as farnesoid X receptor (FXR), vitamin D receptor (VDR), constitutive androstane receptor (CAR), pregnane X receptor (PXR) Takeda G protein-coupled receptor 5 (TGR5) as well as sphingosine-1-phosphate receptor 2 (S1PR2) (Arab et al., 2017; Chiang and Ferrell, 2018). The receptors are expressed not only in enterohepatic tissues but also outside of the liver-gut system to mediate distinct functions throughout the body (Copple and Li, 2016; Chávez-Talavera et al., 2017). Much current knowledge about BAs is concerned with FXR and TGR5 (Arab et al., 2017).

Farnesoid X receptor is activated by different BAs (CDCA > DCA > LCA≫CA) and in turn firmly regulates the synthesis and transport of BAs *via* negative feedback inhibition (de Aguiar Vallim et al., 2013; Wahlström et al., 2016). The binding of BAs to ileal FXR induces the expression of fibroblast growth factor 15/19 (FGF15/19, FGF15 in mice and FGF19 in humans), which binds to the FGF receptor 4 (FGFR4) on the hepatocytes and inhibits the synthesis of BAs (Jia et al., 2018). It is consistent with the fact that the lack of either FGF15/19 or FGFR4 exhibits the increase of hepatic CYP7A1 mRNA and a larger BA pool size (Copple and Li, 2016). Thus, FXR sustains the homeostasis of BAs and avoids the overaccumulation of toxic BAs concentrations in liver cells (Stanimirov et al., 2015). In terms of transport of BAs, ileal FXR activated by unconjugated BAs reduces ASBT expression and causes IBABP and OSTα/β expression (Jia et al., 2018). Therefore, the deficiency of FXR activity induces the disorder of metabolic and host BA regulation (Guzior and Quinn, 2021). As for the other members of the nuclear receptor superfamily, both PXR and VDR are involved in the metabolism and detoxification of BAs and xenobiotics (Chiang, 2009). CAR contributes to the BA homeostasis with PXR and VDR synergistically (Jia et al., 2018).

The expression of TGR5 is different over diverse anatomical compartments, including the liver, gallbladder as well as small and large intestine (Keitel and Häussinger, 2018; Simbrunner et al., 2021). TGR5 shows a higher affinity for secondary BAs than primary BAs and induces disparate intracellular signaling pathways (Sorrentino et al., 2020; Willis et al., 2020). TGR5 is highly expressed in immune cells as well, including macrophages (hepatic Kupffer cells), where BAs induce Toll-like receptor (TLR) signaling (Willis et al., 2020). FXR and TGR5 are reported to colocalize in enteroendocrine cells, and FXR enables TGR5 to induce glucagon-like peptide-1 (GLP-1) secretion, thus stimulating pancreatic β-cells to secrete insulin and connecting BAs to glucose metabolism (Chiang and Ferrell, 2019). In the ileum and colon, another member of the G protein-coupled receptor superfamily, S1PR2, is expressed and activated by conjugated primary BAs (GCA, TCA, GCDCA, and TCDCA) to regulate inflammation, cancer development, and some liver diseases (Guo et al., 2016a; Fiorucci et al., 2021). S1PR2 triggers proliferation *via* ERK1/2 and AKT signaling pathways, which are crucial in the regulation of glucose and lipid metabolism in the liver (Yu et al., 2018). Therefore, regulated expression of BA signaling receptors is essential for hepatic homeostasis.

### Interactions Between Gut Microbiota and Bile Acids

#### Bacteriostasis of Bile Acids

Bile salts are effective antibacterial agents in the intestine that inhibit the overgrowth of bacteria and protect a healthy intestinal tract (Betrapally et al., 2016; Sannasiddappa et al., 2017). Apparent leakage of intracellular potassium from *Staphylococcus aureus* cells was detected after being exposed to bile salts (Sannasiddappa et al., 2017). Furthermore, *Spirochaetes, Gonococcus*, and *Meningococcus* were reported susceptible to BAs (Stacey and Webb, 1947). Both conjugated and unconjugated BAs were demonstrated to inhibit *Escherichia coli, Klebsiella* spp., and *Enterococcus* spp., while unconjugated BAs were found to have stronger antibacterial capability than conjugated BAs when analyzed with *S. aureus*, several *Lactobacillus* species and *Bifidobacterium* species (Sung et al., 1993; Kurdi et al., 2006). Unconjugated primary BAs can disturb membranes and trigger intracellular damage (Guzior and Quinn, 2021). It is because the pK_*a*_ of unconjugated BAs is between 5 and 6.5, however, the conjugation of glycine and taurine lowers pKa and ionizes the conjugates at a neutral pH, leading to the difficulty to cross cell membranes (Sannasiddappa et al., 2017; Guzior and Quinn, 2021; Poland and Flynn, 2021). In addition, antibacterial secondary BAs limit the growth of BAs-intolerant bacteria *via* breaking the cell membrane integrity of bacteria (Kanmani et al., 2020). Moreover, BAs were proved to be entero-protective, possibly *via* their detergent properties and *via* FXR activation, which prevents bacterial over proliferation in the distal small intestine (Betrapally et al., 2016). Thus, the size and composition of the BA pool are regarded as vital factors in regulating the structure of GM (Dahiya et al., 2017).

#### Gut Microbiota and Its Effect on Bile Acids

The adult colon is estimated to contain 200 grams of bacteria. Most bacteria colonizing the gut are obligate anaerobes, while facultatively anaerobic bacteria, archaea and yeast are less abundant (Ridlon et al., 2014). The composition and function of microbiota are affected by diverse host and environmental factors including circadian rhythm, lifestyle, and geographic location (Kolodziejczyk et al., 2019). Seminal studies led to the discovery of the role of GM in regulating the synthesis and metabolism of BAs by adjusting their biomolecular structure.

Glyco-conjugated and tauro-conjugated CA and CDCA are deconjugated in the intestine because anaerobic microbiota can transform conjugated BAs to the unconjugated BAs by bile salt hydrolases (BSHs), and these free BAs are able to trigger BA signaling receptors (Chiang, 2009; Wu et al., 2021). Many bacteria can hydrolyze bile salts (Horác̆ková et al., 2018). For example, choloylglycine hydrolase, a BSH hydrolyzing the amide bond between glycine/taurine and the steroid nucleus of BAs, exits in bacterial genera *Lactobacillus, Bifidobacterium, Clostridium*, and *Bacteroides* (Horác̆ková et al., 2018).

The activity of 7α-dehydroxylase in GM removes the 7α-hydroxy group to produce DCA and LCA. The gene encoding 7α-dehydroxylase is present in *Clostridium scindens* (Chiang, 2009; Jia and Jeon, 2019; Guzior and Quinn, 2021). BSH-rich bacteria from the genera *Enterobacter, Clostridium*, and *Enterococcus* are able to form more secondary BAs (Jia et al., 2018). In humans, approximately 2% of CDCA is converted to its 7β epimer, ursodeoxycholic acid (UDCA), a highly soluble and non-toxic secondary BA formed *via* bacterial 7β-hydroxysteroid dehydrogenase (7β-HSDH) (Chiang and Ferrell, 2019). The activities of BSH and 7α/β-HSDH are abundant in the genera *Clostridium, Bifidobacterium, Enterococcus, Lactobacillus, Listeria*, and *Bacteroides* of the phylum *Firmicutes* and *Bacteroidetes*. Thus, GM above regulates the synthesis of secondary BAs (Chiang and Ferrell, 2019). Furthermore, HSDHs are present in various GM which can oxidize or reduce the hydroxy groups at the 3-, 7-, and 12- carbons of BAs (Ridlon et al., 2016). The differential composition of BAs in germ-free mice and conventional mice demonstrated that GM not only balances the metabolism of secondary BAs, but also hinders BA synthesis by alleviating the inhibition of FXR in the ileum (Sayin et al., 2013). A recent prospective trial identified that a high-fat diet could cause slight increases in the total BAs, especially DCA in plasma and taurodeoxycholate in liver tissues, along with the elevated abundance of *Blautia, Coprococcus, Intestinimonas, Lactococcus, Roseburia*, and *Ruminococcus* (Lin et al., 2019).

In addition to the known transformations of BAs by GM (deconjugation, dehydroxylation, oxidation, desulfation, dehydrogenation, and epimerization), anabolic reactions suggest that the microbiome conjugates CA by unique amino acids, thereby creating newly identified BAs (phenylalanocholic acid, tyrosocholic acid, and leucocholic acid) (Quinn et al., 2020). Although the novel finding indeed deepens our understanding of BAs diversity in our gut, the specific implications for the host and the microbial dynamics are not fully understood (Guzior and Quinn, 2021).

## Gut Microbiota in the Pathogenesis of Non-alcoholic Fatty Liver Disease

### The Pathogenesis of Non-alcoholic Fatty Liver Disease

Non-alcoholic fatty liver disease is defined as steatosis, which influences >5% of hepatic cells and is accompanied by IR with a lack of excessive alcohol intake (Li et al., 2019). Steatosis refers to either large droplets or a mixture of large and small droplets (Bedossa, 2017). Nine to twenty percent of early-stage NASH patients may develop cirrhosis within 5–10 years, and part of them continue to develop HCC (F3/F4-staged fibrosis patients have about 20% incidence of HCC within 5 years) (Bessone et al., 2019). NAFLD is related to many features of the metabolic syndrome, including IR, obesity, hyperlipidemia, and hypertension, and increases the possibility of cardiovascular disease and type 2 diabetes mellitus, but the pathogenesis of NAFLD is complex and not fully understood (Hrncir et al., 2021). It is currently believed that NAFLD is caused by complex interactions of genetic susceptibility, IR, environmental factors, and GM disorders, in addition to the well-known “two-hit” theory or the “parallel multiple-hit theory” (Kolodziejczyk et al., 2019; Zhou and Fan, 2019; Hrncir et al., 2021). In the two-hit theory, the first “hit” includes aberrant triglyceride accumulation and glucose metabolism in hepatocytes, and the second “hit” refers to the proinflammatory cytokines induced by bacterial endotoxin and oxidative stress relevant to reactive oxygen species (Li et al., 2019). According to the “parallel multiple hit theory,” IR is the “first hit” that results in elevated hepatocellular free fatty acids, a major pathogenic factor that makes the liver more vulnerable to further hits, including mitochondrial dysfunction caused by oxidative stress, endoplasmic reticulum stress, TLR4-dependent inflammatory cytokine release, and iron overload (Bessone et al., 2019).

### The Roles of Gut Microbiota in Non-alcoholic Fatty Liver Disease

Recent studies suggest that the compositional and functional diversity of GM may play a role in the progression of NAFLD, as GM diversity in NAFLD patients remains lower than that in healthy subjects (Tilg et al., 2016; Shen et al., 2017; Hrncir et al., 2021). The GM in patients of NAFLD and advanced diseases detected in different studies are listed in Table 1, categorized by phylum, family, and genus. Increased levels of intestinal permeability and inflammation were observed in NAFLD patients with GM dysbiosis. Higher abundances of inflammation-associated *Streptococcus* and *Lactobacillus, Anaerobacter*, and *Escherichia*, and decreased abundances of *Ruminococcaceae* and *Faecalibacterium prausnitzii* were observed in NAFLD patients (Jiang et al., 2015; Duarte et al., 2018). Abnormal GM composition caused by factors such as lifestyle, diet, medication, and environment can alter the immune system and the integrity of intestinal mucosa directly and regulate the levels of inflammatory cytokines and fatty acid oxidation, which contributes to liver diseases (Leung et al., 2016; Zheng et al., 2018; Hrncir et al., 2021).

**TABLE 1:** Differential gut microbiota between patients with different progression of non-alcoholic fatty liver disease.

Patients	Composition	Sex (female/male)	Age (years)	Sequencing method	Phylum	Family	Genus	References
NAFLD vs. healthy controls (HC)	39 participants including 13 NAFLD patients and 26 HC	NAFLD: 7/6 HC: 12/14	NAFLD: 13.6 ± 3.0 HC: 13.3 ± 2.7	16S rRNA	*Epsilonproteobacteria, Gammaproteobacteria↑*	/	*Prevotella↑*	Michail et al., 2015
NAFLD vs. HC	72 participants including 18 NAFLD patients and 54 HC	NAFLD: 12/6 HC: 39/15	NAFLD: 54.0 ± 14.9 HC: 45.9 ± 19.9	16S rRNA	/	*Rikenellaceae, Mogibacterium, Peptostreptococcaceae*↓	*Streptococcus, Bacillus, Lactococcus↑* and *Catenibacterium, Pseudomonas*↓	Caussy et al., 2019
NAFLD vs. HC	85 participants including 53 NAFLD patients and 32 HC	NAFLD: 27/26 HC: 27/5	NAFLD: 48 (22–72) HC: 41 (26–52)	16S rRNA	*Lentisphaerae*↓	*Ruminococcaceae*↓	*Alternatively, Escherichia, Anaerobacter, Lactobacillus, Streptococcus, Clostridium XI?* and *Alistipes, Prevotella, Oscillibacter, Odoribacter, Flavonifractor*↓	Jiang et al., 2015
NAFLD vs. HC	60 participants including 30 NAFLD patients and 30 HC	NAFLD: 17/13 HC: 17/13	NAFLD: 49 (34–57) HC: 51 (47–56)	Multitag pyrosequencing	*Lachnospiraceae↑* and *Ruminococcaceae* ↓	/	*Lactobacillus, Dorea, Robinsoniella, Roseburia↑* and *Oscillibacter* ↓	Raman et al., 2013
NAFLD vs. HC	126 participants including 43 NAFLD patients and 83 HC	NAFLD: 7/36 HC: 13/70	NAFLD: 47 (34.5–61.0) HC: 40.5 (33.0–52.0)	16S rRNA	*Bacteroidetes↑* and *Firmicutes*↓	/	*Coprococcu, Pseudobutyrivibrio, Moryella, Roseburia, Anaerosporobacter, Anaerotruncus, Ruminococcus, Lactobacillus*↓	Wang et al., 2016
NAFLD vs. HC	81 participants including 27 NAFLD patients and 54 HC	NAFLD: 6/21 HC: 31/23	NAFLD: 12.04 ± 2.78 HC: 10.24 ± 2.51	16S rRNA	*Actinobacteria↑* and *Bacteroidetes*↓	*Rikenellaceae*↓	*Bradyrhizobium, Anaerococcus, Peptoniphilus, Propionibacterium acnes, Dorea, Ruminococcus↑* and *Oscillospira*↓	Del Chierico et al., 2017
NAFLD vs. HC	47 participants including 25 NAFLD patients and 22 HC	NAFLD: 6/19 HC: 5/17	NAFLD: 45.5 ± 10.1 HC: 50.5 ± 9.5	16S rDNA	*Proteobacteria, Fusobacteria↑* and *Bacteroidetes*↓	*Lachnospiraceae, Enterobacteriaceae, Erysipelotrichaceae, Streptococcaceae↑* and *Prevotellaceae, Ruminococcaceae*↓	*Escherichia Shigella, Lachnospiraceae Incertae Sedis, Blautia↑* and *Prevotella*↓	Shen et al., 2017
NASH vs. HC	38 participants including 16 NASH patients and 22 HC	NASH: 7/9 HC: 13/9	NASH: 51 ± 9 HC: 44 ± 10	16S rRNA	/	/	*Parabacteroides, Allisonella↑ and Faecalibacterium, Anaerosporobacter*↓	Wong et al., 2013
NASH vs. HC	39 participants including 22 NASH patients and 17 HC	NASH: 12/10 HC: 7/10	NASH: 47 (29–68) HC: 36 (23–58)	qPCR	Percentage of *Bacteroidetes*↓	/	/	Mouzaki et al., 2013
NASH vs. HC	80 participants including 26 NASH patients and 54 HC	NASH: 15/11 HC: 31/23	NASH: 12.27 ± 2.47 HC: 10.24 ± 2.51	16S rRNA	/	/	*Dorea, Ruminococcus, Blautia↑* and *Oscillospira*↓	Del Chierico et al., 2017
NASH vs. HC	28 participants including 6 NASH patients and 22 HC	NASH: not listed HC: 5/17	NASH: not listed HC: 50.5 ± 9.5	16S rDNA	/	*Lachnospiraceae↑*	*Blautia↑*	Shen et al., 2017
NASH vs. HC	38 participants including 22 NASH patients and 16 HC	NASH: 10/13 HC: 6/10	NASH: 13.6 ± 3.5 HC: 14.4 ± 1.8	16S rRNA	*Bacteroidetes, Proteobacteria↑* and *Firmicutes*↓	*Prevotellaceae, Alcaligenaceae, Enterobacteriaceae↑* and *Bifidobacteriaceae, Rikenellaceae, Ruminococcaceae, Lachnospiraceae*↓	*Prevotella, Peptoniphilus↑* and *Bifidobacterium, Oscillospira, Roseburia, Alistipes, Blautia, Coprococcus, Ruminococcus*↓	Zhu et al., 2013
NASH vs. HC	30 participants including 10 NASH and 20 HC	NASH: 4/6 HC: 7/13	NASH: 61 (52–70) HC: 55 (47–64)	16S rRNA	/	/	*Bacteroides↑* and *Prevotella*↓	Boursier et al., 2016
Fibrosis vs. HC	47 participants including 27 fibrosis patients and 20 HC	Fibrosis: 12/15 HC: 7/13	Fibrosis: 62 (56–67) HC: 55 (47–64)	16S rRNA	/	/	*Ruminococcus, Bacteroides↑* and *Prevotella*↓	Boursier et al., 2016
Fibrosis vs. NAFLD	86 participants including 14 fibrosis patients and 72 NAFLD patients	Fibrosis: 12/2 NAFLD: 36/36	Fibrosis: 63.4 ± 3 NAFLD: 49.3 ± 12.6	Whole-genome shotgun sequencing	*Proteobacteria↑* and *Firmicutes*↓	/	/	Loomba et al., 2017
Cirrhosis vs. HC	80 participants including 26 cirrhotic patients and 54 HC	Cirrhosis: 20/6 HC: 39/15	Cirrhosis: 65.1 ± 9.8 HC: 45.9 ± 19.9	16S rRNA	/	*Enterobacteriaceae↑* and *Rikenellaceae, Mogibacterium, Peptostreptococcaceae*↓	*Streptococcus, Megasphaera, Gallibacterium↑ and Catenibacterium, Pseudomonas*↓	Caussy et al., 2019
Cirrhosis vs. HC	181 participants including 98 cirrhotic patients and 83 HC	Cirrhosis: 33/65 HC: 35/48	Cirrhosis: 50 ± 11 HC: 42 ± 9	/	*Proteobacteria, Fusobacteria↑* and *Bacteroidetes*↓	/	*Veillonella, Streptococcus, Clostridium, Prevotella↑* and *Bacteroides, Eubacterium, Alistipes*↓	Qin et al., 2014
Cirrhosis vs. HC	61 participants including 47 cirrhotic patients and 14 HC	Early cirrhosis: 6/17 Advanced cirrhosis: 4/20 HC: 4/10	Early cirrhosis: 55 ± 2 Advanced cirrhosis: 54 ± 5 HC: 52 ± 5	Multi-tagged pyrosequencing	/	*Enterobacteriaceae, Veillonellaceae↑* and *Lachonospiraceae, Ruminococcaceae*↓	*Blautia*↓	Kakiyama et al., 2013
Cirrhosis vs. HC	60 participants including 36 cirrhotic patients and 24 HC	Cirrhosis: 11/25 HC: 10/14	Cirrhosis: 49 ± 11 HC: 46 ± 8	16S rRNA	*Proteobacteria, Fusobacteria↑* and *Bacteroidetes*↓	*Enterobacteriaceae, Veillonellaceae, Streptococcaceae↑* and *Lachnospiraceae*↓	/	Chen et al., 2011
Cirrhosis vs. HC	244 participants including 219 cirrhotic patients and 25 HC	Compensated Outpatients: 92/29 Decompensated Outpatients: 40/14 Inpatients: 31/13 HC: 17/8	Compensated Outpatients: 57.5 ± 6.1 Decompensated Outpatients: 56.8 ± 6.8 Inpatients: 55.9 ± 6.7 HC: 55.7 ± 8.5	Multi-tagged pyrosequencing	/	*Staphylococcae, Enterococceae, Enterobacteriaceae↑* and *Clostridiales XIV, Lachnospiraceae, Ruminococcaceae, Rikenellaceae*↓	/	Bajaj et al., 2014
Cirrhosis vs. HC	58 participants including 30 cirrhotic patients and 28 HC	Cirrhosis: 7/23 HC: 6/22	Cirrhosis: 49 ± 8 HC: 52 ± 9	16S rRNA	/	/	*Veillonella, Megasphaera, Dialister, Atopobium, Prevotella↑* and *Neisseria, Haemophilus, SR1 genera incertae sedis*↓	Chen et al., 2016
Cirrhosis vs. HC	40 participants including 20 cirrhotic patients and 20 HC	Cirrhosis: 8/12 HC: 11/9	Cirrhosis: 60.62 ± 10.46 HC: 60 ± 14	16S rRNA	*Proteobacteria, Bacteroidetes, Cyanobacteria↑* and *Verrucomicrobia, Tenericutes, Euryarchaeota*↓	*Enterobacteriaceae, Lactobacillaceae, Pasteurellaceae, Rikenellaceae, Prevotellaceae, Bacteroidaceae, Porphyromonadaceae, Barnesillaceae, Streptococcaceae, Enterococcaceae, Veillonellaceae↑* and *Verrucomicrobiaceae, Methanobacteriaceae*↓	*Lactobacillus, Haemophilus, Klebsiella, Prevotella, Parabacteroides, Phascolarctobacterium, Veillonella, Enterococcus, Pseudomonas, Streptococcus, Bacteroides, Atopobium, Dialister, Ruminococcus, Christensenella↑* and *Akkermansia, Methanobrevibacter*↓	Ponziani et al., 2019
HCC vs. Cirrhosis	41 participants including 21 HCC patients and 20 cirrhotic patients	HCC: 3/18 Cirrhosis: 8/12	HCC: 66.38 ± 6.67 Cirrhosis: 60.62 ± 10.46	16S rRNA	*Bacteroidetes↑*	*Bacteroidaceae, Streptococcaceae, Enterococcaceae, Gemellaceae↑* and *Verrucomicrobiaceae, Bifidobacteriaceae*↓	*Phascolarctobcterium, Enterococcus, Streptococcus, Gemella, Bilophila↑* and *Akkermansia, Bifidobacterium, Dialister, Collinsella, Adlercreutzia*↓	Ponziani et al., 2019

**FIGURE 2:**
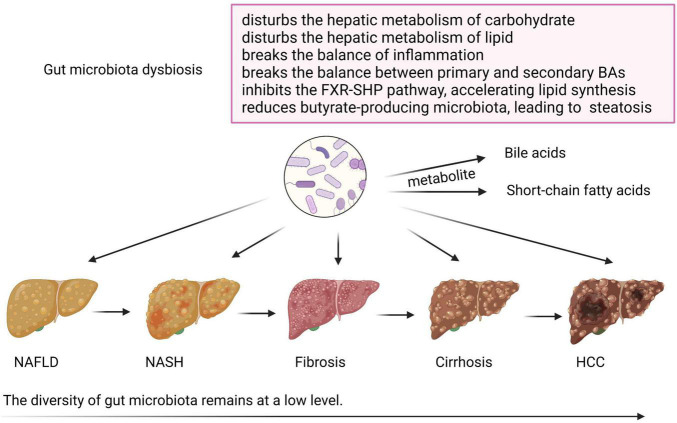
The role of gut microbiota dysbiosis in non-alcoholic fatty liver disease (NAFLD) and its advanced disease progression. During the progression of NAFLD, the diversity of gut microbiota remains at a low level. Gut microbiota dysbiosis disturbs the hepatic metabolism of carbohydrates and lipids and breaks the balance of inflammation. Disturbed gut microbiota also changes the normal metabolism of bile acids (BAs) and short-chain fatty acids, and may cause NAFLD *via* breaking the balance between primary and secondary BAs, inhibiting the FXR-SHP pathway, which accelerates lipid synthesis, and reducing butyrate-producing microbiota, which causes steatosis. Created with BioRender.com.

The role of GM dysbiosis in NAFLD and its advanced disease progression is shown in Figure 2. GM regulates glucose and lipid metabolism through intestinal metabolites, including BAs and short-chain fatty acids (SCFAs), which are altered and involved in the pathogenesis of NAFLD (Chen and Vitetta, 2020; Hu et al., 2020). Normal GM produces 50–100 mmol/l per day of SCFAs, including acetate, propionate, and butyrate, which are produced by fermentation from fibers, balancing lipid metabolism, and stimulating incretin hormone production (Leung et al., 2016; Schoeler and Caesar, 2019). Altered synthesis of multiple SCFAs due to carbohydrate consumption and gut dysbiosis may contribute to NAFLD through multiple mechanisms (Leung et al., 2016). Butyrate is a relevant energy source for colonocytes, and plays an important role in anti-inflammation, so a reduction in the butyrate-producing microbiota in the gut may further cause hepatic steatosis (Park et al., 2016). Besides, a positive correlation was found between the DNA of *Lactobacillus gasseri* and *Lactobacillus taiwanensis* and lipid droplets in the liver (Mokhtari et al., 2017). In conclusion, GM and its metabolites influence the hepatic metabolism of carbohydrates and lipids, as well as the balance of inflammation, thereby affecting NAFLD and its progression (Kolodziejczyk et al., 2019).

### Treatment of Non-alcoholic Fatty Liver Disease *via* Gut Microbiota

It was reported in laboratory research and clinical applications that the disturbed GM structure can be improved by administration of antibiotics, supplement probiotics and prebiotics, or operation of fecal microbial transplantation (FMT) (Hu et al., 2020). For example, the administration of prebiotics can improve body weight, lipid metabolism, IR, and chronic inflammation by modulating GM (Sun et al., 2018). *Akkermansia muciniphila*, a major propionate-producing bacterium, is recommended as a probiotic and an essential gut symbiont to maintain the metabolism homeostasis and alleviate obesity, diabetes, and liver diseases (Mokhtari et al., 2017; Wang et al., 2019). FMT, a GM-altering approach, has been reported to reduce high-fat diet-induced steatosis, increase insulin sensitivity in patients with metabolic syndrome and improve the cognitive performance in cirrhotic patients (Zhou et al., 2017; Moreno-Gonzalez and Beraza, 2021). Thus, studying the role of GM in NAFLD and the effect of specific intestinal flora in NAFLD patients will further help to diagnose and stratify patients and find therapeutic targets (Kolodziejczyk et al., 2019).

## Gut Microbiota-Bile Acids Axis in the Progression of Non-alcoholic Fatty Liver Disease

### Gut Microbiota-Bile Acids Axis in Non-alcoholic Fatty Liver Disease

#### Composition of Bile Acids Altered by Gut Microbiota Leads to Liver Diseases

The composition of the BA pool shows great plasticity because of the modification by GM, thus affecting digestion, nuclear receptor binding, bile fluid toxicity, and solubility, which displays considerable differences between normal individuals and patients with liver diseases (Simbrunner et al., 2021). Due to their cytolytic effects, BAs may be highly toxic when accumulating at high concentrations in the liver (Wu et al., 2021). The hydrophobicity of BAs, which is influenced by the type of conjugation, and the number, orientation, and position of hydroxyl groups, is an important determinant of the toxicity of BAs, and a hydrophobic BA pool is more efficient for intestinal cholesterol absorption (Perez and Briz, 2009; Chávez-Talavera et al., 2017; Molinaro et al., 2018). In addition, excessive secondary BAs were demonstrated to induce reactive oxygen and defective function of mitochondria, leading to DNA apoptosis of cells in the liver (Wu et al., 2021). Therefore, analyzing how GM-modified BAs modulate the host is conducive to understanding the pathogenesis of liver diseases.

#### Receptors of Bile Acids Affect Non-alcoholic Fatty Liver Disease

Bile acids have been revealed as signaling molecules, most likely to induce NAFLD through FXR and TGR5 (Shao et al., 2021). A recent study of methionine and choline-deficient diet-induced mouse model of NASH showed that hepatic FXR signaling was inhibited and CYP7A1 mRNA was upregulated. In contrast, a study concluded that BAs deconjugated by GM induced FXR signaling in the gut, decreased CYP7A1 expression, and inhibited the FXR-small heterodimer partner (SHP) pathway, resulting in accelerated lipid synthesis, and even liver disease (Park et al., 2016). FXR is highly expressed in tissues involved in the enterohepatic circulation of BAs, with the highest expression in the ileum, so dysbiosis of GM may break the balance between primary and secondary BAs, resulting in the interruption of FXR signaling and leading to far-reaching metabolic consequences (Schaap et al., 2014; Hrncir et al., 2021). Elevated ratio of secondary to primary BAs (DCA/CDCA) is known to dysregulate lipid and glucose metabolism by FXR through various target genes (Abdelmalek, 2021; Hrncir et al., 2021).

In the biliary tract, TGR5 expression is critical to prevent BAs overload (Holter et al., 2020). It not only regulates glucose homeostasis and body weight by regulating the expression of GLP-1, but also contributes to anti-inflammatory effects (Wang et al., 2019; Rao et al., 2020). Secondary BAs bind to TGR5 and inhibit NLRP3 inflammasome activation and inflammatory cytokines production, including interleukin-1β (IL-1β) and IL-18 *via* the TGR5-cAMP-PKA axis, thereby improving insulin and glucose tolerance (Guo et al., 2016b; Perino et al., 2021).

### Gut Microbiota-Bile Acids Axis in Non-alcoholic Steatohepatitis

The diagnosis of NASH is different from NAFLD, requiring the presence of steatosis, hepatocellular ballooning, and lobular inflammation with a certain degree of fibrosis on liver biopsy (Friedman et al., 2018). Liver inflammation causes a positive-feedback mechanism that affects BA synthesis (Ridlon et al., 2013). The levels of total BAs, CA, and CDCA in fecal and the levels of GCA, GCDCA, and TCA in plasma were significantly higher in NASH patients compared with those of healthy individuals (Kalhan et al., 2011; Mouzaki et al., 2016). According to a recent study, alterations in the expression of BAs metabolizing enzymes and transporters resulted in an increase in total BAs in plasma, thereby protecting from the hepatotoxicity during the progression to NASH, but such alterations may contribute to the development of liver fibrosis (Suga et al., 2019). The activated FXR helps converting cholesterol into BAs, storing glucose, reducing fat accumulation and inflammation, and preventing hepatic fibrosis. Impaired FXR activation contributes to a lower level of FGF15/19. Drugs targeting the FXR-FGF15/19 axis are efficacious in the treatment of NASH (Abdelmalek, 2021; Hrncir et al., 2021). Obeticholic acid (6α-ethyl-chenodeoxycholic acid) is a semi-synthetic analogue of CDCA approved by the FDA to treat primary biliary cholangitis (Pellicciari et al., 2002). It can bind to the FXR with high affinity to regulate BA synthesis and transport, modulate hepatic lipid, carbohydrate metabolism and immune function, and improve the histological features of NASH effectively (Tølbøl et al., 2018; Hrncir et al., 2021). However, several dose-dependent side effects such as pruritus, increased cholesterol and LDL along with lower HDL in plasma were observed in NASH patients treated with an FXR agonist (Fiorucci et al., 2020). INT-767, a dual FXR and TGR5 agonist, potently increases intracellular Ca^2+^ level, cAMP concentration, and GLP-1 secretion and regulates gene expression involved in the BA synthesis, thus it exerts potent therapeutic benefits against NASH (Pathak et al., 2017; Wang et al., 2018).

The impairment of the intestinal epithelial barrier is an early event in NASH pathogenesis (Mouries et al., 2019). Proinflammatory cytokines, including TNF-α, IL-6, and IFN-γ were increased in the gut mucosa biopsies (Jiang et al., 2015). Proinflammatory bacteria are consistently associated with gut permeability, leading to the transportation of bacterial components to the liver, accelerating simple steatosis and the progression to NASH (Schnabl and Brenner, 2014; Jiang et al., 2015). At the phylum level, *Bacteroides* was increased in abundance in NASH patients, compared to healthy controls (Kolodziejczyk et al., 2019). Members of the genus *Escherichia* have been reported to be elevated in NASH patients, increasing blood ethanol concentrations (Zhu et al., 2013). Later studies found that endogenous alcohol produced by *Escherichia coli* increases gut permeability (Jiang et al., 2015).

### Gut Microbiota-Bile Acids Axis in Liver Fibrosis

Fibrosis is the consequence of a long-time process of hepatocyte damage and is divided into four stages, F0 to F4 (Shen et al., 2017; Marra and Svegliati-Baroni, 2018). Fibrosis is induced by resident cells in the liver, including hepatocytes, Kupffer cells, and hepatic stellate cells (HSCs) (Kanmani et al., 2020). An earlier experiment demonstrated that a higher ratio of secondary to primary BAs decreased and higher conjugated cholate increased the likelihood of significant fibrosis (*F* ≥ 2) (Puri et al., 2018). Contrary to the results of this experiment, recent studies have showed that the abundance of total BAs in the plasma of fibrotic individuals is higher, which is positively correlated with the stage of exacerbation of fibrosis due to higher levels of primary BAs rather than secondary BAs (Nimer et al., 2021). Taurodeoxycholate and glycodeoxycholate, which are conjugated 12a-hydroxylated (12a-OH) BAs, were found to increase in mice with liver fibrosis due to their activation of HSCs (Xie et al., 2021).

Compositions of GM in fibrosis patients were determined in a prospective study *via* whole-genome shotgun sequencing of DNA extracted from stool samples and revealed that the abundance of *Firmicutes* was higher in moderate NAFLD while *Proteobacteria* was higher in advanced fibrosis (Loomba et al., 2017). Another study determined the composition of GM in stool samples by 16S ribosomal RNA gene sequencing, demonstrating that the abundance of *Bacteroides* and *Ruminococcus* was increased in fibrosis patients, whereas *Prevotella* abundance was decreased (Boursier et al., 2016). In several studies, researchers found that specific intestinal bacteria are correlated with fibrosis severity and primary BAs, such as *Ruminococcus bromii, F. prausnitzii, Roseburia intestinalis*, and *Megamonas* (Lee et al., 2020). For example, *F. prausnitzii*, one of the butyrate-producing bacteria, has the ability of immunomodulation and is highly sensitive to a slight increase in bile salts concentrations (Duarte et al., 2018; Bromke and Krzystek-Korpacka, 2021). Moreover, UDCA treatment may increase the abundance of *F. prausnitzii* and *Megamonas* (Liu et al., 2020). Sterilization of the gut led to less bacteria-derived LPS in plasma, which might ameliorate liver fibrosis in mice, thus improving intestinal disorder with non-absorbable antibiotics or strengthening the intestinal barrier may ameliorate liver fibrosis (Mazagova et al., 2015; Kanmani et al., 2020).

### Gut Microbiota-Bile Acids Axis in Liver Cirrhosis

Decreased conversion of primary to secondary BAs in feces is more common in patients with cirrhosis, which is associated with the abundance of certain GMs (Kakiyama et al., 2013). In a recent review on BAs, TCA, GCA, TCDCA, and GCDCA were described as BA biomarkers in liver cirrhosis (Yang et al., 2019). Some species in *Ruminococcaceae* and *Blautia* are known to produce 7α-dehydroxylase, leading to higher DCA levels in the stool of patients with cirrhosis (Lin et al., 2019). Changes in the ratio of *Bacteroides/Firmicutes* and elevation of Gram-negative bacteria are pathogenic (Kakiyama et al., 2013; Shao et al., 2021). Compared with healthy controls, patients with cirrhosis had fewer *Bacteroidetes* and *Firmicutes* but higher abundances of *Proteobacteria* and *Fusobacteria* (Chen et al., 2011; Qin et al., 2014). Moreover, studies have shown that pathogenic *Enterobacteriaceae* as well as strains from oral microbiota, such as *Veillonella* and *Streptococcus* were enriched in patients with cirrhosis, consistent with the spread of bacteria from the mouth to the gut (Chen et al., 2011; Qin et al., 2014; Yu and Schwabe, 2017). The GM composition of patients with cirrhosis showed relatively lower levels of *Lachnospiraceae, Ruminococcaceae, Clostridialies XIV*, and *F. prausnitzii* and a higher abundance of *Bacteroidaceae* (Bajaj et al., 2014; Aron-Wisnewsky et al., 2020a; Moreno-Gonzalez and Beraza, 2021). The microbial signature of cirrhosis showed high abundance of *Megasphaera, Dialister, Atopobium, Prevotella*, and *Gallibacterium* (Chen et al., 2016; Caussy et al., 2019).

### Gut Microbiota-Bile Acids Axis in Hepatocellular Carcinoma

Hepatocellular carcinoma is one of the top three causes of cancer mortality worldwide (Schwabe and Greten, 2020). TLR4 activation is involved in hepatocarcinogenesis through HSCs, macrophages, and hepatocytes, resulting in a chronic inflammatory state. TLR4 also promotes fibrosis and induces the expression of epiregulin in HSCs, a potent HCC-promoting hepatocyte mitogen (Schwabe and Greten, 2020). TLR4 activation and GM-induced proliferation are required for HCC promotion, and gut sterilization targeting advanced stages of hepatocarcinogenesis may ameliorate HCC symptoms (Dapito et al., 2012). Compared with NASH-cirrhotic patients, NASH-HCC patients have a distinct GM composition, accompanied by reduced diversity but elevated abundances of anti-inflammatory-associated bacteria, *Bifidobacterium* and *Blautia*, and higher levels of *Enterococcus, Ruminococcus, Bacteroides, Phascolarctobacterium*, and *Oscillospira* (Moreno-Gonzalez and Beraza, 2021). In a recent study, *Lactobacilli* was found to be associated with BAs levels in serum samples and liver injury in NASH-HCC patients (Sydor et al., 2020).

Changes in BAs may cause metabolic disorders, hepatic lesions, resistance to apoptosis, and high proliferation, which contribute to tumorigenesis (Wu et al., 2021). A preclinical rodent model has shown that the toxic effects of secondary BAs transformed by GM led to the development of HCC (Moreno-Gonzalez and Beraza, 2021). For example, in HSCs, the enterohepatic circulation of DCA, synergizing with the TLR2 agonist, lipoteichoic acid, a component of Gram-positive gut microbes, induces the senescence-associated secretory phenotype, which suppresses anti-tumor immunity and promotes tumor-promoting factors, such as cyclooxygenase-2 (COX-2), followed by COX-2-mediated prostaglandin E2 (PGE2) inhibition of antitumor immunity through PGE2 receptor subtype 4 (EP4), resulting in HCC in mice (Yoshimoto et al., 2013; Loo et al., 2017). Excessive COX-2 expression and PGE2 production were also detected in HSCs of HCC patients without cirrhosis and NASH (Loo et al., 2017). It was demonstrated in a NASH-HCC model that the secondary BAs accumulation affected by GM also induces carcinogenesis through mTOR signaling activation in hepatocytes (Yamada et al., 2018). Furthermore, the expression of CXCL16 may affect the abundance of CDCA in non-tumor liver tissues of HCC patients, while it is negatively correlated with the secondary BA, glycolithocholic acid (Ma et al., 2018). Mice depleted of Gram-positive bacteria with vancomycin showed accumulation of hepatic natural killer T (NKT) cells and reduction in HCC. However, administration of secondary BAs or colonization of bile acid–metabolizing bacteria reversed NKT cell accumulation and tumor growth (Ma et al., 2018). In the same research, *Clostridium scindens* colonization was found to mediate BA conversion and reduce hepatic NKT cells by suppressing CXCL16 expression in hepatic sinusoidal endothelial cells to affect the growth of HCC (Ma et al., 2018). Rifaximin, a non-absorbable antibiotic, may target the gut–liver axis and moderately reduce HCC development (Dapito et al., 2012; Yu and Schwabe, 2017).

## Conclusion

Growing evidence suggests that BAs and GM play critical roles in the pathogenesis of NAFLD. BAs are endogenous molecules and therapeutic targets in liver diseases, effectively maintain cholesterol and lipid homeostasis in the gut, regulate metabolic signaling through multiple receptors, and act as the intermedium of complex molecular crosstalk between humans and their gut microbiota (Suga et al., 2019). GM intervenes in the pathogenesis of NAFLD by transporting its substances or metabolites (Ji et al., 2019). In the gut, interactions between GM and BAs contribute to the development of disease. Further studies of the GM-BAs axis are necessary to explore the pathogenic mechanism of metabolic diseases, such as NAFLD. Currently, most studies on the GM-BAs axis are based on mouse models, yet there is a vast chasm between humans and mice in the immune system, BAs metabolism, GM, and etc. Thus studies taken on humans are important for a better understanding of the role of GM-BAs axis in the progression of NAFLD (Shao et al., 2021). The gut-liver axis has attracted much attention in the studies of metabolic diseases, and the prevention and treatment of NAFLD targeting the gut-liver axis may be imperative in the future (Ji et al., 2020). The complex roles of BAs and the host microbiome in NAFLD are just beginning to be understood. More molecular mechanisms and signaling pathways for the prevention, diagnosis and treatment of NAFLD need to be validated and applied clinically.

## Author Contributions

YN and ML edited the manuscript. YX, QW, XYG, and YL searched the references. TXZ, CX, and TZ prepared the figures and table. X-JG and MZ were responsible for the supervision of the manuscript. All authors read and approved the final manuscript.

## Conflict of Interest

The authors declare that the research was conducted in the absence of any commercial or financial relationships that could be construed as a potential conflict of interest.

## Publisher’s Note

All claims expressed in this article are solely those of the authors and do not necessarily represent those of their affiliated organizations, or those of the publisher, the editors and the reviewers. Any product that may be evaluated in this article, or claim that may be made by its manufacturer, is not guaranteed or endorsed by the publisher.

## Abbreviations

NAFLD: non-alcoholic fatty liver disease
IR: insulin resistance
NASH: non-alcoholic steatohepatitis
HCC: hepatocellular carcinoma
BAs: bile acids
GM: gut microbiota
CYP7A1: cholesterol 7α-hydroxylase
C4: 7α-hydroxy-4-cholesten-3-one
CA: cholic acid
CDCA: chenodeoxycholic acid
CYP8B1: sterol 12α-hydroxylase
CYP27A1: sterol 27-hydroxylase
CYP7B1: oxysterol 7α-hydroxylase
BSEP: bile salt export pump
ASBT: apical sodium-dependent bile acid transporter
IBABP: ileal bile acid-binding protein
OST: organic solute transporter
NTCP: sodium taurocholate co-transporting polypeptide
LCA: lithocholic acid
DCA: deoxycholic acid
FXR: farnesoid X receptor
VDR: vitamin D receptor
CAR: constitutive androstane receptor
PXR: pregnane X receptor
TGR5: Takeda G protein-coupled receptor 5
S1PR2: sphingosine-1-phosphate receptor 2
SHP: small heterodimer partner
FGF15/19: fibroblast growth factor 15/19
FGFR4: fibroblast growth factor receptor 4
TLR: toll-like receptor
GLP-1: glucagon-like peptide-1
BSHs: bile salt hydrolases
UDCA: ursodeoxycholic acid
7β-HSDH: 7β-hydroxysteroid dehydrogenase
SCFAs: short-chain fatty acid
FMT: fecal microbial transplantation
HSCs: hepatic stellate cells
COX-2: cyclooxygenase-2
PGE2: prostaglandin E2
EP4: PGE2 receptor subtype
NKT: natural killer T.
